# From Basic Science to Clinical Practice: The Role of Cancerous Inhibitor of Protein Phosphatase 2A (CIP2A)/p90 in Cancer

**DOI:** 10.3389/fgene.2023.1110656

**Published:** 2023-02-24

**Authors:** Beibei Chen, Huihui Hu, Xiaobing Chen

**Affiliations:** ^1^ Department of Medical Oncology, Affiliated Cancer Hospital of Zhengzhou University, Henan Cancer Hospital, Zhengzhou, Henan, China; ^2^ Zhengzhou Key Laboratory for Precision Therapy of Gastrointestinal Cancer, Zhengzhou, Henan, China

**Keywords:** CIP2A/p90, cancer, tumor-associated antigen (TAA), signaling pathways, biomarker, prognosis

## Abstract

Cancerous inhibitor of protein phosphatase 2A (CIP2A), initially reported as a tumor-associated antigen (known as p90), is highly expressed in most solid and hematological tumors. The interaction of CIP2A/p90, protein phosphatase 2A (PP2A), and c-Myc can hinder the function of PP2A toward c-Myc S62 induction, thus stabilizing c-Myc protein, which represents a potential role of CIP2A/p90 in tumorigeneses such as cell proliferation, invasion, and migration, as well as cancer drug resistance. The signaling pathways and regulation networks of CIP2A/p90 are complex and not yet fully understood. Many previous studies have also demonstrated that CIP2A/p90 can be used as a potential therapeutic cancer target. In addition, the autoantibody against CIP2A/p90 in sera may be used as a promising biomarker in the diagnosis of certain types of cancer. In this Review, we focus on recent advances relating to CIP2A/p90 and their implications for future research.

## 1 Introduction

The sera of patients diagnosed with cancer contain antibodies that can react with a unique group of autologous cellular proteins called tumor-associated antigens (TAAs) (Chen et al., 2018). The immune system of cancer patients is a sensor of alterations in the structure and/or function of participants in tumorigenesis pathways and is capable of immune responses in the form of autoantibodies against these TAAs (Jhunjhunwala et al., 2021). Circulating autoantibodies have been used as ’probes’ in cancer patients to isolate TAAs, which have been shown to be cellular factors participating in known tumorigenesis pathways (Tan, 2001; Tan and Zhang, 2008; Zhang et al., 2022). The constitution of TAAs do not include all cellular antigens identified by autoantibodies in cancer sera as some autoantibodies may exist in conditions that pre-date malignancy. Thus, many approaches aimed at identifying and characterizing authentic TAAs have been identified by anti-TAA autoantibodies, which can be used as biomarkers for diagnosis or early detection only after extensive evaluation with cancer and non-cancer sera (Zhang and Tan, 2010; Li et al., 2021).

CIP2A was initially identified as a TAA and was named p90 due to its molecular weight of 90 kDa (Soo Hoo et al., 2002). Autoantibodies against p90 were found in 21% of sera from a group of patients with liver cancer. Sera with anti-p90 localized to the cytoplasm were detected by indirect immunofluorescent staining in fetal mouse liver but not in adult liver (Zhang et al., 2002). Full-length cDNA encoding p90 was successfully isolated from a T24 expression library, including a sequence coding for a 905-amino-acid protein, predicted to have a molecular mass of 102 kDa. In a subsequent study, p90 was found to be identical to cancerous inhibitor of protein phosphatase2A (CIP2A) by a research group from Finland (Junttila et al., 2007). The function of CIP2A/p90 is related to its binding with c-Myc and inhibiting dephosphorylation of S62 caused by PP2A (Farrington et al., 2020).

Many studies have focused on the function of CIP2A/p90 since the protein was identified by our study group. This review focuses on recent advances, which have primarily been associated with the determination of CIP2A/p90 function or its potential as a biomarker for the early detection of various types of cancer.

## 2 The function of CIP2A/p90 in cancers

Protein kinase phosphorylation and protein phosphatase (PP) dephosphorylation are considered the most common mechanisms involved in intracellular protein regulation and signal transduction. Their imbalance is associated with cystic fibrosis, Alzheimer’s disease (AD), and other diseases, such as cancer (Ruvolo, 2019; Shentu et al., 2019; Mercier et al., 2020; Khan M M et al., 2021; Vainonen et al., 2021). According to the dephosphorylated amino acid residues, PP has been categorized into two families, the protein tyrosine phosphatase family and the serine threonine phosphatase family. PP2A is a widely conserved serine threonine phosphatase and has been defined as a kind of tumor suppressor protein (Chen et al., 2013; Perrotti and Neviani, 2013). PP2A is a trimeric holoenzyme, with a scaffold A subunit, a catalytic C subunit, and several different regulatory B subunits. The B subunits determine the subcellular localization and substrate specificity of the PP2A holoenzyme (Ruvolo, 2016). Although PP2A has multiple substrates, its anti-cancer function is mostly related to the dephosphorylation and stabilization of c-Myc (Pippa and Odero, 2020). Recent studies had shown that PP2A is widely involved in the regulation of cellular physiological and pathological processes, such as energy metabolism, cell cycle, DNA replication, proliferation, apoptosis, and inflammatory responses (Sangodkar et al., 2016; Baskaran and Velmurugan, 2018; Kauko and Westermarck, 2018; Remmerie and Janssens, 2019; Khan R et al., 2021). C-Myc is overexpressed in most cancers as a transcription factor with oncogenic capability that mediates cell proliferation, apoptosis, differentiation, adhesion, migration, metabolism, and DNA replication (Sun and Gao, 2017; Duffy et al., 2021; Dhanasekaran et al., 2022; Grieb and Eischen, 2022). As mentioned earlier, CIP2A, encoded by the *KIAA1524* gene located on human chromosome 3q13.13, is a major endogenous PP2A-inhibiting protein. The interaction among CIP2A/p90, PP2A, and c-Myc can hinder the function of PP2A toward c-Myc S62 induction and therefore stabilize c-Myc protein, which represents a potential role of CIP2A/p90 in the promotion of cancer (Pippa and Odero, 2020; Scarpa et al., 2021).

CIP2A/p90 plays an important role in the proliferation, apoptosis, invasion, migration, epithelial–mesenchymal transition (EMT), cell cycle, and drug resistance of different tumor cells. CIP2A/p90 was overexpressed in 65%–90% of tissues in almost all human cancers, and this has been associated with poor survival (Tarek et al., 2021). The molecular mechanism of CIP2A/p90 in cancer has mostly been associated with the interaction among CIP2A/p90, PP2A, and c-Myc (Table 1). On the other hand, several studies have indicated that the silencing of CIP2A/p90 by small interfering RNAs (siRNA) inhibited the growth of xenografted tumors of various kinds of cancer cells (Table 1).

**TABLE 1:** siRNA downregulates CIP2A on tumor cells and potential molecular mechanisms.

Type of cancer	Cell lines	Proliferation	Apoptosis	Invasion	Migration	EMT	Cell cycle	Drug resistance	Potential molecular mechanisms	References
Head and neck squamous cell cancer (HNSCC)	UT-SCC-7	↓^a^	—^b^	—	—	—	—	—	c-Myc ↓	Junttila et al. (2007)
	UT-SCC-9									
	CAL27, FaDu	↓	↑^d^	—	—	—	—	—	Axin2 ↓, MMP7 ↓, c-Myc ↓	Kleszcz et al. (2019)
Nasopharyngeal carcinoma (NPC)	CNE-2, SUNE-1	↓	—	—	—	—	—	—	c-Myc ↓	Liu et al. (2014a)
Neuroblastoma	SK-N-AS, SK-N-BE, SH-EP, WAC2	—	—	—	↓	—	—	—	—	Williams et al. (2019)
Oral cancer	NCI-60	↓	—	↓	↓	—	—	—	c-Myc ↓	Jung et al. (2013)
	SCC-25	↓	—	—	—	—	—	—	—	Cantini et al. (2013)
Non-small-cell lung cancer (NSCLC)	H1299	↓	—	═^c^	—	—	—	—	AKT-mTOR signaling pathway	Dong et al. (2011); Lei et al. (2014)
	L78	↓	—	—	—	—	—	—	—	Ma et al. (2011)
	SPCA1	↓	—	—	—	—	—	↓(Cisplatin)	AKT signaling pathway	Ma et al. (2011); Wei et al. (2014)
	A549	↓	—	═	—	—	—	↓(Cisplatin)	AKT signaling pathway	Dong et al. (2011); Ma et al. (2011); Wei et al. (2014)
Breast cancer	MDA-MB-231	↓	—	↓	—	—	↓	—	PP2A/c-Myc/p27Kip1 signaling pathway	Xing et al. (2016); Liu et al. (2017b)
	BT549									
	MCF-7/ADR	↓	↑	—	—	—	—	—	—	Zhu and Wei (2021)
Esophageal squamous cell cancer	EC109	↓	↑	—	—	—	═	—	c-Myc ↓	Qu et al. (2012)
Gastric cancer	MKN-28	↓	—	—	—	—	—	—	c-Myc ↓	Khanna et al. (2009)
	KATOIII									
	SGC7901/DDP	↓	↑	—	—	—	—	↓(Cisplatin)	—	Ji et al. (2018)
Hepatocellular carcinoma (HCC)	Hep3B	↓	↑	—	—	—	↓	—	CDK2↓, CDK4↓	Yang et al. (2018)
	HepG2								Cyclin D1↓	
	SMMC-7721									
	BEL-7402									
	MHCC97H	↓	—	↓	↓	—	—	—	—	Li et al. (2022)
	SNU387									
Colon cancer	Caco-2	↓	—	—	—	—	—	—	ERK ↓	Chen J.S et al. (2015)
	HCT116	↓	—	—	—	—	—	—	c-Myc ↓	Wiegering et al. (2013)
	HT29	↓	—	—	—	—	—	↓(5-fluorouracil, oxaliplatin, SN38)	—	Teng et al. (2012)
	HCT116 SW480	↓	—	—	—	—	↓	—	—	Denk et al. (2021)
	LS174t									
Pancreatic cancer	SW1990	↓	—	—	—	—	—	↓(Gemcitabine)	BCL2 ↓, AKT ↓	Xu et al. (2016)
Clear cell renal cancer	786-O	—	—	↓	—	↓	—	—	—	Tang et al. (2015)
	A498	═	—	↓	↓	—	—	—	c-Myc ↓	Ren et al. (2011)
	KRC/Y									
	Caki-1	↓	—	—	—	—	—	—	AKT signaling pathway	Gao et al. (2020)
Prostate cancer	LNCaP	↓	—	—	—	—	—	—	—	Khanna et al. (2015)
	PC-3	—	—	—	—	—	↓	—	CIP2A interacts with Sgol1	Pallai et al. (2015)
	C4-2	↓	—	—	—	—	—	↓(Cabazitaxel)	—	Huang et al. (2015)
Bladder cancer	T24	↓	↑	↓	↓	↓	—	—	—	Xue et al. (2013); Pang et al. (2016)
Cervical cancer	HeLa	↓	—	—	—	↓	—	↓(Doxorubicin, cisplatin, and paclitaxel)	c-Myc ↓, Pgp ↓, MEK/ERK signaling pathway (CIP2A interacts with H-Ras)	Liu W et al. (2011); Wu et al. (2015); Liu J et al. (2016)
Endometrioid adenocarcinoma (EAC)	SiHa	↓	—	—	—	—	—	—	c-Myc ↓	Liu J et al. (2011)
	Caski									
	Ishikawa	↓	↑	↓	↓	—	↓	—	c-Myc ↓, Cyclin D1↓	Yu et al. (2018)
	An3ca	↓	—	↓	↓	—	↓	—	c-Myc ↓, Cyclin D1↓	Yu et al. (2018)
Ovarian cancer	SKOV3^DDP^	↓	—	—	—	—	—	↓(Cisplatin)	AKT signaling pathway	Zhang et al. (2015)
	A2780, SKOV3	↓	—	—	—	—	↓	↓(Paclitaxel)	Cyclin D1 ↓, c-Myc ↓, p-Rb ↓, Bcl-2 ↓, p-AKT ↓	Fang et al. (2012)
Astrocytoma	A172	↓	↑	—	—	—	—	—	c-Myc ↓, pAKT ↓, BCL2 ↓	Yi et al. (2013)
	U87									
Melanoma	FEMX1, WM1366, WM983b, WM9	↓	↑	—	—	—	—	—	PI3K/AKT signaling pathway	Flørenes et al. (2015)
	A375	—	—	↓	↓	—	—	—	—	Shi et al. (2014)
Osteosarcoma	MG-63	↓	—	↓	—	—	—	—	c-Myc ↓, pAKT ↓	Zhai et al. (2014)
Glioblastoma	U251MG, WK1	↓	—	—	—	—	—	—	—	Khanna et al. (2020)
Colorectal cancer	DLD1, HT29	↓	↑	—	—	—	—	—	c-Myc ↓	Chen et al. (2020)
Multiple myeloma (MM)	RPMI-8226, NCI-H929	↓	↑	—	—	—	—	—	c-Myc ↓, PI3K/AKT/mTOR signaling pathway	Yang et al. (2016); Zheng et al. (2016)
Acute myeloid leukemia (AML)	HEL	↓	—	—	—	—	—	—	c-Myc ↓	Barragán et al. (2015)
	HL60	↓	—	—	—	—	—	—	—	Wang et al. (2011)
Chronic myelocytic leukemia (CML)	K562	↓	↑	—	—	—	—	—	c-Myc ↓	Wang H. W et al. (2014)

^a^ Inhibition or downregulation.

^b^ Unknown.

^c^ No significant effect.

^d^ Promotion or upregulation.

As shown in Table 1, silencing CIP2A/p90 with siRNA can further reduce the expression of c-Myc to inhibit cell proliferation and induce cell apoptosis (Yang et al., 2016; Zheng et al., 2016). In addition, siRNA inhibition of CIP2A transcription can make colorectal cancer cells sensitive to radiation and reduce their survival rate *in vitro* (Birkman et al., 2018). CIP2A/p90 can promote p27Kip1 phosphorylation at Ser10 by *via* inhibiting Akt-associated PP2A activity, which seems to relocalize p27Kip1 to the cytoplasm. On the other hand, CIP2A/p90 can also recruit c-Myc to mediate the transcriptional inhibition of p27Kip1 and induce cell cycle arrest at the G2/M phase (Liu H et al., 2017). In addition, in cells expressing human papillomavirus 16 oncoprotein E6, it can promote the transformation of the G1/S cell cycle through B-Myb (Tian et al., 2018). Furthermore, several studies have shown that CIP2A/P90 regulates STAT3 phosphorylation and IL-17 expression in Th17 cells by regulating the intensity of interaction between AGK and STAT3 (Chen et al., 2013; Khan et al., 2020a; Khan et al., 2020b). However, only a few studies on the molecular mechanism of the CIP2A/p90 regulating function are mentioned aboved. CIP2A/p90 also has a PP2A-independent function, which can directly interact with Polo-like kinase1 (PLK1) but not with mitosis gene A-related kinase 2 (NEK2), H-Ras, etc., to regulate cellular function. CIP2A/p90 can interact with PLK1 and enhance the stability and activity of PLK1, thereby promoting mitosis in human cancer cells (Kim et al., 2013). The depletion of CIP2A/p90 may also prolong cell division time. CIP2A/p90 interacts with NEK2 during the G2/M phase, and can facilitate centrosome separation and mitotic spindle dynamics in cell cycle progression (Jeong et al., 2014). CIP2A/p90, in association with the oncogene *H-Ras* and through the recruitment of the MEK/ERK signaling pathway and c-Myc dephosphorylation by PP2A, is required for EMT in the progression of cancer (Wu et al., 2015). Patients with both HOXB13 T and CIP2A T alleles have a higher risk of prostate cancer and invasive disease, earlier biochemical recurrence, and lower disease-specific life expectancy. HOXB13 protein binding to the *CIP2A* gene can functionally promote CIP2A transcription (Sipeky et al., 2018). Studies have confirmed that *CIP2A* is an essential gene in BRCA1 and BRCA2 mutant cells, finding that the CIP2A-TOPBP1 axis can protect chromosome stability, which is a synthetic lethal target for BRCA mutant cancer (Adam et al., 2021).

## 3 The signaling pathways and regulation network of CIP2A/p90

**FIGURE 1:**
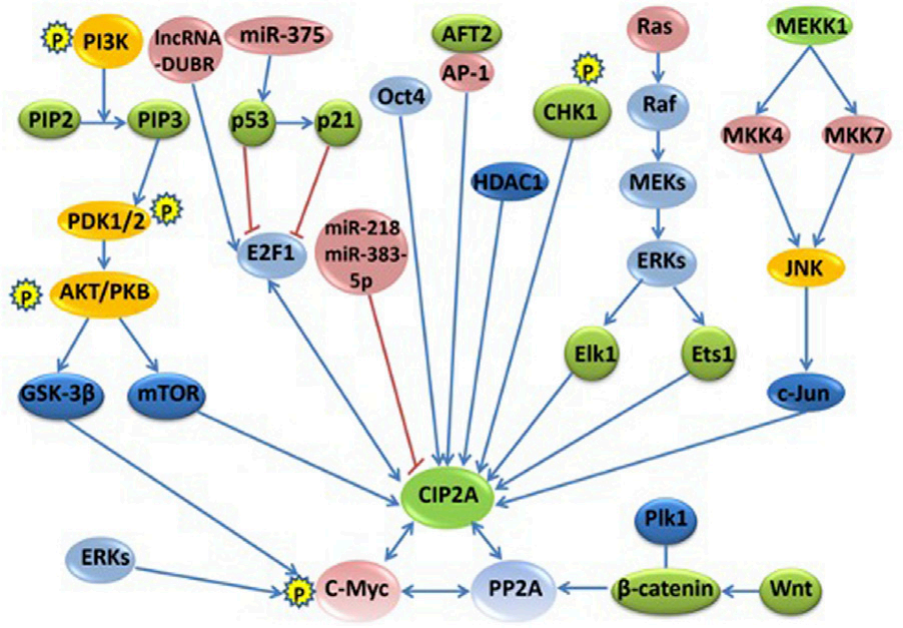
The signaling pathways and regulation networks of CIP2A/p90. Several signaling pathways, including the PI3K–AKT–mTOR pathway, the RAS–MEK–ERK pathway, the Wnt–β-catenin pathway, the MKK4/MKK7-JNK-c-Jun pathway, the p53-p21-E2F1-CIP2A/p90 pathway, and the phosphorylation and degradation of c-Myc, non-coding RNAs, and other regulation factors, such as Oct4, AFT2, CHK1, and HDAC1, are included in this figure. Bidirectional blue arrows indicate interactions between two entities; unidirectional blue arrows indicate a positive influence of an entity on another; red lines indicate a negative influence of one entity on another.

The regulation network of CIP2A/p90 was established through direct interactions of CIP2A/p90 or indirectly through interactions of CIP2A/PP2A with either multiple key cellular proteins/transcription factors or with oncogenic signaling pathways. Figure 1 shows the signaling pathways and regulation mechanisms mainly associated with CIP2A/p90.

### 3.1 The PI3K–AKT–mTOR pathway

Phosphatidylinositol 3-kinase (PI3K) is a heterodimer consisting of a regulatory subunit (p85) and a catalytic subunit (p110). Activated PI3K can convert phosphatidylinositol 4,5-bisphosphate (PIP2) to PIP3, which is a second messenger through 3-phosphoinositide-dependent kinase1 (PDK1), indirectly activates AKT. The activated AKT acts on a variety of substrates, such as mTOR and glycogen synthase kinase-3β (GSK-3β), to regulate cell growth, proliferation, and other functions (Vogelstein et al., 2013). IL-10 phosphorylates cAMP response element-binding protein (CREB) through the PI3K/AKT signaling pathway, thereby regulating *CIP2A*/p90 gene expression (Sung et al., 2013). Based on our previous study, it was found that CIP2A/p90 can regulate AKT phosphorylation at S473 under growth factor stimulation. Our research also showed that CIP2A/p90 might promote cell proliferation through the AKT–mTOR signaling pathway (Lei et al., 2014). In addition, a new study further confirmed that the overexpression of CIP2A was a key contributory event of AKT phosphorylation in the correlation analysis of p-AKT and CIP2A in 220 clinical samples, and emphasized that the CIP2A-AKT axis is a promising therapeutic target for breast cancer (Luque et al., 2022).

### 3.2 The RAS–MEK–ERK pathway

Ras, which is stimulated by extracellular signals, recruits Raf to bind and activate it on the cell membrane. The activated Raf (MAPKKK) can reactivate MAPKK, which in turn activates extracellular protein kinases (ERKs) (also known as MAPK), and finally, the activated ERK can further activate a number of transcription factors, such as Elk-1, Ets1, ATF, NF-κB, and c-Myc, to trigger a variety of biological effects (De et al., 2014). Ets1, as the transcription factor, can mediate high CIP2A/p90 expression in human cancers through increased activity of the EGFR-MEK1/2-ERK pathway (Khanna et al., 2011). The binding of Ets1 and Elk1 together to the proximal CIP2A/p90 promoter is absolutely required for CIP2A/p90 expression in liver, endometrial, and cervical carcinoma cells (Pallai et al., 2012). Additionally, 17β-estradiol (E2) activates EGFR, thus stimulating the MEK1/2 and PI3K pathways and further increasing the expression of CIP2A/p90 through the MEK1/2-induced transcription factor Ets1 to enhance the proliferation of cancer cells (Choi et al., 2014).

### 3.3 The MKK4/MKK7-JNK-c-Jun pathway

JNK belongs to the mitogen-activated protein family (MAPK), which responds to certain stimuli, such as cytokines, UV radiation, heat, and osmotic shock. The activated JNK leads to cell migration, proliferation, and invasion in cancers. According to our research, we found that the overexpression of CIP2A/p90 is associated with increased JNK pathway through the phosphorylation of MKK4/MKK7-JNK-c-Jun signaling. However, the exact mechanism by which CIP2A/p90 modulates the JNK phosphorylation pathway is still unknown (Peng et al., 2015). Knockdown of CIP2A decreases JNK phosphorylation and the phosphorylation of downstream transcriptional factors ATF2 and c-Jun, the transcriptional activity of which is also decreased. Furthermore, the expression level of CIP2A also affects the phosphorylation of the upstream kinase of JNK, MKK4/MKK7 (Peng et al., 2015).

### 3.4 The P53-p21-E2F1-CIP2A/p90 pathway

The overexpression of E2F1 leads to activated cell cycle and uncontrolled cellular proliferation in the majority of human cancers. Owing to the inactivation of p53 or p21, the overexpression of E2F1 promotes the expression of oncoprotein CIP2A/p90, which in turn increases stabilizing serine 364 phosphorylation of E2F1. The p53-p21-Rb pathway can negatively regulate the activity of E2F1 transcription (Lucas et al., 2015). Furthermore, research has shown that the positive feedback loop of E2F1-CIP2A/p90 is very important to the sensitivity of senescence and growth arrest induction in breast and cervical cancer cells (Laine et al., 2013; Wang et al., 2017). The CIP2A-AKT-mTOR pathway controls cell growth, apoptosis, and autophagy. Polyphyllin I (PPI) and polyphyllin VII (PPVII) are natural components extracted from *Paris polyphylla* that have anticancer properties. Examination of the mechanism revealed that PPI and PPVII significantly upregulate p53, induce caspase-dependent apoptosis, and suppress the CIP2A-AKT-mTOR pathway. The activation of autophagy is mediated through PPI and PPVII, which induce the inhibition of mTOR (Feng F. et al., 2019).

### 3.5 Non-coding RNA

MicroRNA, with a length of 18–25 nucleotides, is a type of small single non-coding RNA that regulates gene post-transcriptional expression through binding with complementary sequences, which can degrade the target mRNA or inhibit its translation (Jung et al., 2013). miR-218 can bind to the 3'-UTR region of CIP2A/p90 in cutaneous melanoma cells to regulate the gene expression of *CIP2A*/p90. The upregulation of miR-218 inhibits the expression of CIP2A/p90 and meanwhile suppresses the functions of melanoma cells, such as migration, proliferation, invasion, and cell cycle (Lu et al., 2015). The study examined the effect of miR-218 on the expression of CIP2A in clear cell renal cell carcinoma (ccRCC). The results showed that the expression level of miR-218 in ccRCC was lower than that in adjacent non-tumor kidney tissues. The downregulation of CIP2A or the overexpression of miR-218 in ccRCC cells can inhibit cell proliferation and migration (Wei et al., 2019). miR-383-5p directly targets CIP2A/p90 to inhibit cell proliferation by G1 cell cycle phase arrest and promotes apoptosis in lung adenocarcinoma (Zhao et al., 2017). CIP2A/p90 is also targeted by miR-375, which stimulates the expression of p21 due to the promotion of its major transcriptional activator, p53, and consequently restrains the action of CIP2A/p90 and c-Myc in cell proliferation. These findings suggest that microRNA can act as a tumor suppressor of oncogenic elements, such as CIP2A/p90 (Jung et al., 2014). In addition, miR-548b-3p regulates proliferation, apoptosis, and mitochondrial function by targeting CIP2A in HCC (Lin and Wang, 2018). There is an automatic regulation feedback loop between CIP2A and miR-301a. Additionally, the feedback of miR-301a promotes the expression of CIP2A through ERK/CREB signal (Yin et al., 2019). A specificity protein 1 (SP1)-induced long non-coding RNA, DPPA2 upstream binding RNA (DUBR), upregulates CIP2A expression through E2F1-mediated transcription regulation, which also plays a role in upregulating CIP2A at the mRNA level by binding miR-520d-5p as a competing endogenous RNA (Liu et al., 2022). The knockdown of LINC00665 can also significantly decrease the cell proliferation, migration, and invasion of HCC, while overexpression of the short peptides of LINC00665 (CIP2A-BP) can markedly increase cell proliferation, invasion, and migration (Li et al., 2022).

### 3.6 Other regulation factors

The Wnt-β-catenin pathway: after the activation of Wnt, β-catenin is stabilized and bound to the T-cell factor (Tcf)/lymphoid enhancer factor (Lef) family transcription factors, thus leading to a transcriptional activation of target genes (Huang et al., 2019). Aberrant activation of the Wnt/β-catenin pathway is a common event in many types of cancers (Zhang and Wang, 2020). The upregulation of CIP2A/p90 might indirectly lead to reduced β-catenin levels *via* PP2A inactivation, reinforcing the polo-like kinases (Plk1)-dependent β-catenin inhibition (Li et al., 2015). Additionally, CIP2A/p90 enhances the stabilization of β-catenin to promote fibronectin-induced cancer cell proliferation (Gao et al., 2017).

Phosphorylation and degradation of c-Myc: ERK can phosphorylate c-Myc Ser62 to stabilize it. Then, GSK-3β further phosphorylates c-Myc Thr58, followed by prolyl isomerase (PIN-1), which can transform c-Myc (including both Ser62 and Thr58 phosphorylation sites) from a cis-structure to a trans-structure (Posternak and Cole, 2016). PP2A can catalyze the trans-structure of c-Myc Ser62 dephosphorylation to form the trans-structure of c-Myc (including the Thr58 phosphorylation site), which may be further ubiquitinated and degraded by protein ligase complex (containing FWB7) (Dang, 2012). CIP2A/p90 interacts directly with c-Myc and inhibits PP2A activity toward c-Myc Ser 62, thereby preventing c-Myc proteolytic degradation (Junttila et al., 2007).

Other regulation factors also exist. The expression of CIP2A/p90 in various tumor cells is regulated by other regulation factors with a certain complexity and cell specificity. Moreover, most of them are transcription factors. Octamer-binding transcription factor 4 (Oct4) positively regulates the expression of CIP2A/p90 both in embryonic stem cells and testicular cancer cell lines. The co-expression of Oct4 and CIP2A/p90 is also associated with the increased radio-resistance and aggressiveness in HNSCC cell lines (Ventelä et al., 2015). In addition, the study found that CIP2A can directly interact with TopBP1 and coordinate DNA damage-induced mitotic checkpoint and proliferation, thus driving the initiation and progression of basal breast cancer (Laine et al., 2021). In mouse embryonic fibroblasts, the transcription factor ATF2 binds to the AP-1 site in the promoter region of the *CIP2A*/p90 gene and initiates gene transcription (Mathiasen et al., 2012). Activated transcription factor 6 (ATF6) is one of the three major stress transduction factors of the endoplasmic reticulum and has been proven to promote chemotherapy resistance by changing the survival of cancer cells. Recent studies have shown that endoplasmic reticulum stress-related ATF6 upregulates CIP2A/p90, which helps to improve the prognosis of colon cancer (Liu X et al., 2018). The activity of checkpoint kinase 1 (CHK1) promotes the transcription of CIP2A/p90, thereby inhibiting the activity of PP2A, the tumor suppressor. In addition, the phosphorylation of CHK1 can upregulate the expression of the *CIP2A*/p90 gene through phosphorylation of serine 345 of CHK1 *via* DNA damage response kinases (DNA-PK) in human gastric cancer, ovarian cancer, colon cancer, and neuroblastoma (Khanna et al., 2013; Khanna et al., 2020). Histone deacetylase 1 (HDAC1) regulates *CIP2A*/p90 gene expression in colorectal cancer cells. The inhibition of HDAC1 by (S)-2 downregulated the transcription of CIP2A/p90 and unleashed PP2A activity, thereby inducing growth arrest and apoptosis in colorectal cancer cells (Balliu et al., 2016).

## 4 CIP2A/p90 expression and its clinical role in tumors

Compared with normal or para-cancerous tissues, CIP2A/p90 (protein or mRNA) is overexpressed or amplified at a high frequency in the vast majority of solid and hematological tumors (Tang et al., 2018). Recent studies have shown that the aberrant expression level of CIP2A/p90 is either significantly correlated with tumor stages or serves as a prognostic marker for overall survival (OS) and disease-free survival (DFS) (Table 2). According to numerous studies, the high expression of CIP2A/p90 in some cancers, such as cutaneous melanoma, breast cancer, colon cancer, cervical cancer, prostate cancer, and oral cancer, is associated with pathologic high-grade tumor and the progression of disease (Côme et al., 2009; Vaarala et al., 2010; Böckelman et al., 2011a; Böckelman et al., 2012; Shi et al., 2014; Velmurugan et al., 2019). As shown in our previous study, CIP2A/p90 is rarely expressed in non-cancerous/non-transformed cells, but is abundantly expressed in typically transformed cells (Soo Hoo et al., 2002).

**TABLE 2:** Expression of CIP2A in various tumor tissues and its clinical significance.

Type of cancer	Positive rate	Relationship with survival rate	Relationship with prognosis	References
NPC	90.7% (254/280)	+^a^	+	Liu et al. (2014b)
Tongue cancer	97.3% (71/73)	+	+	Böckelman et al. (2011b)
Oral cancer	100% (133/133)	+	+	Velmurugan et al. (2019)
Oral squamous cell carcinoma	54.3% (19/35)	±	±	Alzahrani et al. (2020)
HNSCC	78.6% (11/14)	-^b^	-	Junttila et al. (2007)
	80.8% (42/52)	+	+	Routila et al. (2016)
	82.7% (43/52)	+	-	Ventelä et al. (2015)
Thyroid carcinoma	85.3% (81/95)	+	+	Chao et al. (2016)
Lung cancer	84.7% (61/72)	-	-	Peng et al. (2015)
NSCLC	72.2% (65/90)	+	+	Dong et al. (2011)
	76.3% (74/97)	+	+	Xu et al. (2012)
	88.3% (184/209)	+	+	Cha et al. (2017)
Breast cancer	39.4% (13/33)	+	-	Côme et al. (2009)
	35% (448/1280)	+	+	Yu G et al. (2013)
	46% (565/1228)	-	+	Laine et al. (2013)
	100% (46/46)	-	-	Liu C. Y et al. (2014)
Esophageal squamous cell cancer	90% (36/40)	-	×^c^	Qu et al. (2012)
Esophageal adenocarcinoma	97.3% (110/113)	×	+	Rantanen et al. (2013)
Gastric cancer	65% (145/223)	+	+	Khanna et al. (2009)
	67.6% (25/37)	+	+	Chen F. F et al. (2015)
Esophagogastric junction adenocarcinoma	64.6% (42/65)	+	-	Li et al. (2019)
Colorectal cancer	87.9% (661/752)	×	×	Böckelman et al. (2012)
	100% (167/167)	+	+	Teng et al. (2012)
	89.4% (93/104)	+	+	Wiegering et al. (2013)
	80.7% (21/26)	+	+	Chen et al. (2020)
HCC	100% (136/136)	+	+	He et al. (2012)
	77.9%^d^ (106/136)	+	+	Huang C. Y et al. (2012)
Pancreatic cancer	56.3% (54/96)	+	+	Wang et al. (2013)
	70.8% (51/72)	+	+	Xu et al. (2016)
Cholangiocarcinoma	78.9% (45/57)	+	+	Xu et al. (2013)
Renal cancer	70.1% (75/107)	+	+	Ren et al. (2011)
	50.0% (55/110)	+	+	Wang P et al. (2019)
	73.7%^d^ (59/80)	-	+	Gao et al. (2020)
Bladder cancer	72.6% (85/117)	+	+	Xue et al. (2013)
	78.8% (63/80)	-	-	Pang et al. (2016)
	41.9% (18/43)	-	-	Huang P et al. (2012)
Prostate cancer	72.9% (43/59)	×	×	Vaarala et al. (2010)
	96.2% (101/105)	-	×	Celikden et al. (2020)
Cervical cancer	52.8% (38/72)	-	-	Liu X et al. (2014)
	60.8% (31/51)	-	-	Wu et al. (2015)
Ovarian cancer	82.8% (434/524)	+	+	Böckelman et al. (2011a)
	65.8% (100/152)	-	-	Fang et al. (2012)
Melanoma	100.0% (65/65)	+	+	Shi et al. (2014)
Multiple myeloma	46.3% (19/41)	-	+	Liu et al. (2017c)
Osteosarcoma	76.5% (39/51)	-	-	Zhai et al. (2014)
AML	57.8%^d^ (67/116)	-	-	Wang et al. (2011)
	100%^d^ (203/203)	+	+	Barragán et al. (2015)
CML	75.7%^d^ (56/74)	-	-	Wang J et al. (2014)

^a^ Related.

^b^ Unknown.

^c^ Unrelated.

^d^ CIP2A mRNA, positive rate (the rest was CIP2A protein positive rate).

As shown in Table 2, some controversial conclusions have been made in the same type of cancer by different research groups. Out of two studies (He et al., 2012; Huang C.Y et al., 2012), He et al. concluded that the high expression of CIP2A/p90 can predict poor outcome in patients with hepatocellular carcinoma, and therefore, this can be used as a significant prognostic factor for DFS and OS (He et al., 2012). Conversely, in the study by Huang et al., the expression of intratumoral CIP2A/p90 mRNA was not associated with prognosis, whereas non-cancerous CIP2A/p90 mRNA was shown to be an independent prognostic factor of OS and recurrence-free survival (RFS) (Huang L.P et al., 2012). Therefore, more extensive research evaluating both CIP2A/p90 protein and mRNA expression, with normal controls, is needed. As with hepatocellular carcinoma, the results from three investigations evaluating the prognostic value of CIP2A/p90 expression were contradictory (Böckelman et al., 2012; Teng et al., 2012; Wiegering et al., 2013). The investigations carried out by Wiegering et al. (2013) and Teng et al. (2012), examining 104 and 167 colon cancer specimens, respectively, both revealed that CIP2A/p90 expression is positively associated with prognosis. By contrast, Böckelman et al. (2012) analyzed 752 specimens and showed there was no significant association between CIP2A/p90 expression and prognosis. This disparity might be due to the different size of each sample or the different antibodies used for staining CIP2A/p90. In addition, the high expression of CIP2A/p90 has diagnostic significance in some cancers, such as papillary thyroid carcinoma, breast cancer, and chronic myeloid leukemia (Liu C Y et al., 2014; Chao et al., 2016; Xing et al., 2016; Clark et al., 2021).

## 5 Autoantibody to CIP2A/p90 as biomarker in cancers

As described above , CIP2A/p90 was initially isolated and characterized as a type of TAA (Soo Hoo et al., 2002). The immune system of certain cancer patients can recognize these aberrant TAA proteins as foreign antigens, thus producing antibodies, called autoantibodies in response. Therefore, anti-TAA autoantibodies might be regarded as biomarkers for the early detection of certain types of cancer (Tan, 2001; Tan and Zhang, 2008). According to our previous studies and others, the frequency of autoantibodies to CIP2A/p90 in sera is significantly higher than that of normal controls. When we selected a panel of TAAs, such as CIP2A/p90, the accumulative positive autoantibodies’ reactions in sera were much higher (Shi et al., 2005; Xie et al., 2011; Liu et al., 2014a). Some data showed the selected panel of TAAs had high specificity and sensitivity as immunodiagnostic biomarkers in both he test cohort and the validation cohort (Zhang et al., 2016; Hoshino et al., 2017). In addition, a few of the panel TAAs, including CIP2A/p90, had a high diagnostic performance in the detection of cancers, especially for the patients at early stage (Zhang et al., 2016; Wang X et al., 2019; Table 3).

**TABLE 3:** Frequency of anti-CIP2A/p90 and the TAA panel in cancer patients and normal controls.

Cancer	TAA panel number	Percentage (number) of TAA positivity				References
		CIP2A/p90	Normal controls	Panel	Normal controls	
Prostate	6	30.8% (41/133)	3.1% (3/96)	92.5% (122/133)	14.8% (14/96)	Shi et al. (2005)
Prostate	6	-^a^	-	79% (103/131)	16% (19/121)	Xie et al. (2011)
Breast	-	19.1% (32/168)	2.3% (2/88)	-	-	Liu et al. (2014a)
Breast	5	-	-	38% (147/386)	-	Sumazaki et al. (2021)
ESCC	4	-	-	77.01% (499/648)^b^		Zhang et al. (2016)
				78.49% (292/372)^c^		
Gastric cancer	6	8.0% (8/100)^b^	1.3% (1/79)^b^	49.0% (49/100)^b^	7.6% (6/79)^b^	Hoshino et al. (2017)
		11.3% (28/248)^c^	4.1% (3/74)^c^	52.0% (52/100)^c^	9.5% (7/74)^c^	
Ovarian cancer	9	16.7% (22/132)	2.0% (3/147)	61.4% (81/132)	15.0% (22/147)	Wang X et al. (2019)

^a^ Unknown.

^b^ Test cohort.

^c^ Validation cohort.

The clinical value of the autoantibody responses to CIP2A/p90 and other TAAs might be further validated by more studies of different cancers. The more precise circumscriptions about whether the expression level of anti-TAA autoantibodies varies with disease progression or the response to treatment, and when autoantibodies against these TAAs appear as early predictors of cancers, also needs further investigation (Liu J et al., 2011).

## 6 CIP2A/p90 as a potential therapeutic target in cancers

The overexpression of CIP2A/p90 can upregulate the drug resistance of tumor cells to chemotherapy (Liu et al., 2022). Based on the pathophysiology of cancer cells, it can be suggested that effective therapeutic responses against them require simultaneous inhibition of kinase signaling pathways and the reactivation of their inhibitors, such as PP2A (Soofiyani et al., 2017; Westermarck, 2018). CIP2A/p90 siRNA and some small-molecule compounds can inhibit some tumor cell proliferation and corresponding nude mice xenografts. The inhibition was related to the downregulation of CIP2A/p90, the downstream molecules of which could increase PP2A activity and attenuate AKT phosphorylation (Table 4).

**TABLE 4:** Antitumor research related to CIP2A downregulation.

Compounds	Suppressed tumor cells	Inhibition of nude mice xenografts	Reduce resistance	References
CIP2A siRNA	Human tongue squamous cell carcinoma (SCC) cell line CAL 27	Xenograft model of oral cancer cell CAL27	—^a^	Cantini et al. (2013)
	Bladder cancer cell (T24)	Xenograft model of bladder cancer cell T24	—	Xue et al. (2013)
Lapatinib	Breast cancer cell (HCC 1937; MDA-MB-468/MDA-MB-231)	—	—	Liu J et al. (2016)
Genistein	Breast cancer cell (MCF-7-C3 and T47D)	—	—	Zhao et al. (2016)
Fingolimod	Breast cancer cell (MDA-MB-231and BT-474)	Xenograft model of breast cancer cell MDA-MB-231	—	Zhao et al. (2016)
Tamoxifen	Breast cancer cell (MDA-MB-231, MDA-MB-468, MDA-MB-453, and SK-BR-3)	Xenograft model of breast cancer cell MDA-MB-468	—	Liu N et al. (2014)
Cucurbitacin B	Breast cancer cell (MCF-7/Adr)	—	Doxorubicin	Cai et al. (2016)
	Glioblastoma multiform (GBM) cell, (DBTRG-05MG, U251MG, U118MG, U87MG, and LN229)	Xenograft model of GBM cell U118MG	—	Qin et al. (2018)
	Gastric cancer cell (SGC7901/DDP and SGC7901)	—	Cisplatin	Liu et al. (2017d)
	The t (8:21)-bearing AML cell line kasumi-1, acute promyelocytic leukemia (HL60), acute myelomonocytic leukemia (U937), chronic myelogenous leukemia (K562), and Burkitt’s lymphoma (Raji) and T-cell acute lymphoblastic leukemia (Molt-4)	Xenotransplantation model of AML cell	—	Ma et al. (2019)
	Human gefitinib-resistant NSCLC cell A549, NCI-H1299 (H1299), NCI-H1975 (H1975), NCI-H820 (H820), and human normal lung epithelial cell (16-HBE)	H1975 cell transplantation model	Gefitinib	Liu et al. (2019)
Bortezomib	HNSCC cell (Ca9-22, SAS and SCC-25)	—	—	Lin et al. (2012)
	Breast cancer cell (HCC-1937, MDA-MB-231 and MDA-MB-468)	Xenograft model of breast cancer cell HCC-1937	—	Tseng et al. (2012)
	Colon cancer cell (LoVo)	Xenograft model of colon cancer cell LoVo	—	Ding et al. (2014)
	HCC cell (Sk-Hep1 and Huh-7)	—	Radiation	Huang L.P et al. (2012)
	Cervical cancer cell (SiHa)	(Bortezomib and radiation combination) xenograft model of cervical cancer cell SiHa	Radiation	Huang P et al. (2012)
	Leukemia cell (HL-60 and KG-1)	Xenograft model of leukemia cell HL-60	—	Liu et al. (2013)
	Non-small cell lung cancer cell (HCC4006)	—	Erlotinib	Saafan et al. (2021)
Bortezomib and its derivative	HCC cell (Huh-7, Hep3B and Sk-Hep1)	Xenograft model of HCC cell Huh-7	Anti-death receptor 5 antibodies CS-1008	Chen et al. (2010); Hou et al. (2013)
Carfilzomib	Leukemia cell (HL-60, KG-1, THP-1 and K562)	Xenograft model of Leukemia cell HL-60 and K562	—	Liu et al. (2017a)
Ellagic acid	Lung adenocarcinoma cell HOP62 and H1975 (harboring L858R/T790M EGFR mutation)	Xenograft model of lung cancer cell HOP62	—	Duan et al. (2019)
Polyphyllin I	NSCLC cell A549 and DDP-resistant A549/DDP cells	—	Cisplatin	Feng et al. (2019b)
	GC cell (SGC7901, SGC7901/DDP and GES-1)	Xenograft model of GC cell SGC7901/DDP	Cisplatin	Zhang et al. (2018)
	PC cancer cell (PC3 and DU145)	Xenograft model of PC cell DU145	—	Liu C.Y et al. (2018)
Erlotinib	NSCLC cell (H358)	Xenograft model of NSCLC cell H358	—	Wang C Y et al. (2014)
	HCC (PLC5 and Hep3B)	—	—	Yu H.C et al. (2013)
Erlotinib and its derivative	HCC (Sk-Hep1)	—	—	Chen et al. (2012)
Erlotinib derivative TD-19	NSCLC cell (H460)	Xenograft model of NSCLC cell H460	—	Chao et al. (2014)
Erlotinib derivative TD52	HCC (PLC5, Huh-7, Hep3B, and Sk-Hep1)	Xenograft model of HCC cell PLC5	—	Yu et al. (2014)
	Triple-negative breast cancer (TNBC) cells (HCC-1937)	Xenograft model of TNBC cell MDA-MB-468	—	Liu et al. (2017b)
	MDA-MB-231 and MDA-MB-468)			
Afatinib	NSCLC cell (H358 and H441)	Xenograft model of NSCLC cell H358	—	Chao et al. (2015)
Celastrol	NSCLC cell (H1975 and A549)	Xenograft model of NSCLC cell H1975 and A549	—	Liu et al. (2014b)
	Chondrosarcomas (CS) cell (SW1353 and JJ012)	—	—	Wu et al. (2017)
Ethoxysan-guinarine	NSCLC cell (H1975 and A549)	—	Cisplatin	Liu et al. (2014a)
	CRC cell (SW620, SW480, HT29 and HCT116)	Xenograft model of CRC cells SW620	—	Jin et al. (2018)
Temsirolimus	Colon cancer cell (HCT-15 and SW480)	Temsirolimus and cetuximab combination xenograft model of colon cancer cell HCT-15	Cetuximab	Wang H.W et al. (2014)
Euxanthone	CRC cell (HT29, HCT116, SW620, LOVO and SW480)	Xenograft model of CRC cells HT29	—	Wang et al. (2018)
Gambogenic acid	HCC (Hep G2 and Bel-7402)	—	—	Yu et al. (2016)
Huaier polysaccharide (HP-1)	ccRCC cells (A498 and 786-O)	Xenograft model of ccRCC cells A498	Sunitinib	Fang et al. (2019)
FTY720	Neuroblastoma cell SK-N-AS (CRL-2137), SK-N-BE (2) (CRL-2271), SH-EP and WAC (2)	Xenograft model of neuroblastoma cells SH-EP and WAC (2)	—	Williams et al. (2019)
	Medulloblastoma cell (D341, D384, and D425)	Xenograft model of medulloblastoma cells D341, D384, and D425	—	Garner et al. (2018)
2,5-Dimethyl Celecoxib	Glioblastoma cell (LN229, A172, U251 and U87MG)	Xenotransplantation model of glioblastoma cell LN229 cells in nude mice	—	Gao et al. (2021)
Polyphyllins I and VII	NSCLC cell (A549 and A549/DDP)	—	Cisplatin	Feng et al. (2019b)
(+)-Cyanidan-3-ol	Squamous cell skin cancer (SCSC) cell (A431)	DMBA/TPA-induced SCSC and xenograft model of SCSC cell A431	—	Monga et al. (2022)
		A431		

^a^ Unknown.

According to Table 4, the mechanism by which some small-molecule compounds downregulate CIP2A/p90 has been elucidated. Hypoglycemia and metformin impair the metabolic plasticity and growth of tumors by regulating the PP2A-GSK3b-MCL-1 axis (Elgendy et al., 2019). Lapatinib, erlotinib derivative TD52, and afatinib interfered transcription factor Elk1 combined with the CIP2A/p90 promoter further downregulate the expression of CIP2A/p90 separately in breast cancer cells, liver cancer cells, and NSCLC cells (Yu et al., 2014; Chao et al., 2015; Liu C Y et al., 2016; Liu et al., 2017a). Bortezomib, as a proteasome inhibitor, has an anti-tumor effect in HCC, HNSCC, leukemia, breast cancer, and colon cancer by inhibiting the CIP2A-PP2A-AKT signaling pathway (Chen et al., 2010; Lin et al., 2012; Tseng et al., 2012; Liu et al., 2013; Ding et al., 2014). Celastrol, bound to CIP2A/p90 in NSCLC cells, promotes the connection of CIP2A/p90 with the carboxyl terminus of Hsp70-interacting protein (CHIP) and induces the degradation of CIP2A/p90 (Liu et al., 2014b). Gambogenic acid induces the degradation of CIP2A/p90 through the ubiquitin–proteasome pathway in HCC cells (Yu et al., 2016). Notably, the direct and accurate antagonists of CIP2A/p90 are still unknown. There are multiple challenges in establishing direct CIP2A/p90-target drugs as effective clinical anticancer therapies.

## 7 Conclusion

CIP2A/p90 is overexpressed in most types of cancer and is positively correlated with the poor prognosis of many patients. The interaction among CIP2A/p90, PP2A, and c-Myc is an important mechanism of CIP2A/p90 in promoting cancer. Owing to the nature of CIP2A/p90, which can play important roles in the proliferation, apoptosis, invasion, migration, EMT, cell cycle, and drug resistance of tumor cells, it can be used as a potential diagnostic biomarker, as well as an antitumor drug target. However, there are still some important issues to be resolved: (1) the function of CIP2A/p90 in both cell proliferation and drug resistance suggests that it plays an important role in cancer stem cells, which have drug resistance and rapid proliferation. (2) The signaling pathways and regulation networks of CIP2A/p90 are complex. Genomic or systems-level analysis with new tools and technologies will reveal how the signaling pathways and regulators of CIP2A/p90 contribute to tumorigenesis. (3) The precise molecular structure of CIP2A/p90 has not yet been resolved. Therefore, the direct antagonists of CIP2A/p90 still need further investigation and additional application in clinical therapy. (4) The clinical value of autoantibody against CIP2A/p90 as biomarker in cancer needs to be further clinically validated. Overall, there is an urgent need for large studies that will clearly validate the clinical significance of CIP2A/p90, the potential benefit of which is huge.
